# Exploring the intricate relationship between IL-1β and IL-18 in the context of osteoarthritis

**DOI:** 10.3389/fimmu.2026.1870390

**Published:** 2026-07-23

**Authors:** Anjali Kumari, Mahaboobkhan Rasool

**Affiliations:** Immunopathology Lab, School of Biosciences and Technology, Vellore Institute of Technology (VIT), Vellore, Tamil Nādu, India

**Keywords:** cartilage degeneration, chondrocytes, interleukin-18, interleukin-1β, osteoarthritis, senescence

## Abstract

Interleukin-1β (IL-1β) and interleukin-18 (IL-18) are key members of the IL-1 cytokine family that contribute to the initiation and progression of osteoarthritis (OA). Although both cytokines share structural similarities and use MyD88-dependent signaling pathways, accumulating evidence indicates that they function complementarily rather than redundantly. IL-1β acts as an early catalyst for catabolic processes, leading to cartilage breakdown and cellular senescence, whereas IL-18 serves as an inflammasome-dependent enhancer that activates the immune response. This review summarizes the roles of IL-1β and IL-18 at multiple biological levels in OA progression and discusses their secretion and activation mechanisms. Additionally, we explore current and emerging pathological strategies that target the effects of IL-1β and IL-18 on resident cells during knee OA progression. Despite substantial evidence suggesting that cytokines are involved in OA progression, IL-1β-targeted therapies have shown limited clinical success, highlighting the need to explore additional targets. By integrating multiple pathological mechanisms, this review proposes a conceptual framework in which OA progression comprises three interconnected phases: cytokine-driven initiation, amplification, and chronicity, all linked to senescence. In conclusion, this review evaluates current pharmacological strategies. It underscores future research directions that focus on key elements of the IL-1β/IL-18 signaling pathway, emphasizing the significance of biomarker-driven and combination therapies for improving OA management.

## 1 Introduction

Osteoarthritis (OA) is the most common degenerative condition worldwide, involving multiple joint tissues, including articular cartilage, synovium, subchondral bone, menisci, ligaments, and the fat pads, all of which contribute to disease initiation and progression and undergo important biomechanical and inflammatory changes through the activation of the innate immune system (1).OA has affected nearly 500 million people globally, with the knee joint being the most frequently affected site. The major risk factors for disease initiation are aging, obesity, mechanical injury, genetic changes, and chronic low-grade inflammation (2). This immune system activation underscores the importance of understanding the role of inflammatory cytokines, as they are key mediators in cartilage and in the downstream signaling pathways that contribute to disease progression (3).

The IL-1 family in humans and most mammals includes 11 cytokines, some of which promote inflammation and others reduce it. These IL-1 cytokines are proteins weighing 17–18 kDa, characterized by a β-trefoil pyramidal barrel structure composed of six two-stranded β-hairpins (4). The IL-1 family members include IL-1α, IL-1β, IL-18, IL-33, IL-36α, IL-36β, IL-36γ, IL-1Ra, IL-36Ra, IL-37, and IL-38. These cytokines play important roles in the pathogenesis of arthritic disease progression (5). These cytokines play an important role in maintaining bone and cartilage homeostasis by modulating the biology of chondrocytes, osteoblasts, and synovial cells. Among them, interleukin-1β (IL-1β) and interleukin-18 (IL-18) are upstream activators due to their strong pro-catabolic effects. Both cytokines initiate autocrine and paracrine inflammatory loops, where IL-1β enhances its own production and acts as a mediator of IL-6 and matrix metalloproteinases (MMPs) (6). IL-18 shares structural resemblance with IL-1β, which is why it is classified as part of the IL-1 superfamily. This cytokine primarily originates from an inactive precursor that is converted to functional IL-18 via enzymatic cleavage (6). Initially, IL-18, which is upregulated by NK and T cells, was identified as promoting interferon-γ (IFN-γ) production, either alone or in conjunction with IL-12. This led to its designation as an IFN-γ-inducing factor, thereby rapidly activating monocytes and macrophages (7).

IL-18 directly affects the cartilage as chondrocytes and osteoblasts secrete it. *In vitro* and *in situ* findings suggest that IL-18 promotes cartilage degradation by increasing the expression of COX-2, iNOS, and MMPs in chondrocytes and stimulates the production of IL-6 and nitric oxide, which promotes matrix degradation (7). Consequently, IL-18 creates a harmful environment within the cartilage, characterized by an excess of degradative enzymes and reduced repair capacity (7).

**Figure 1:**
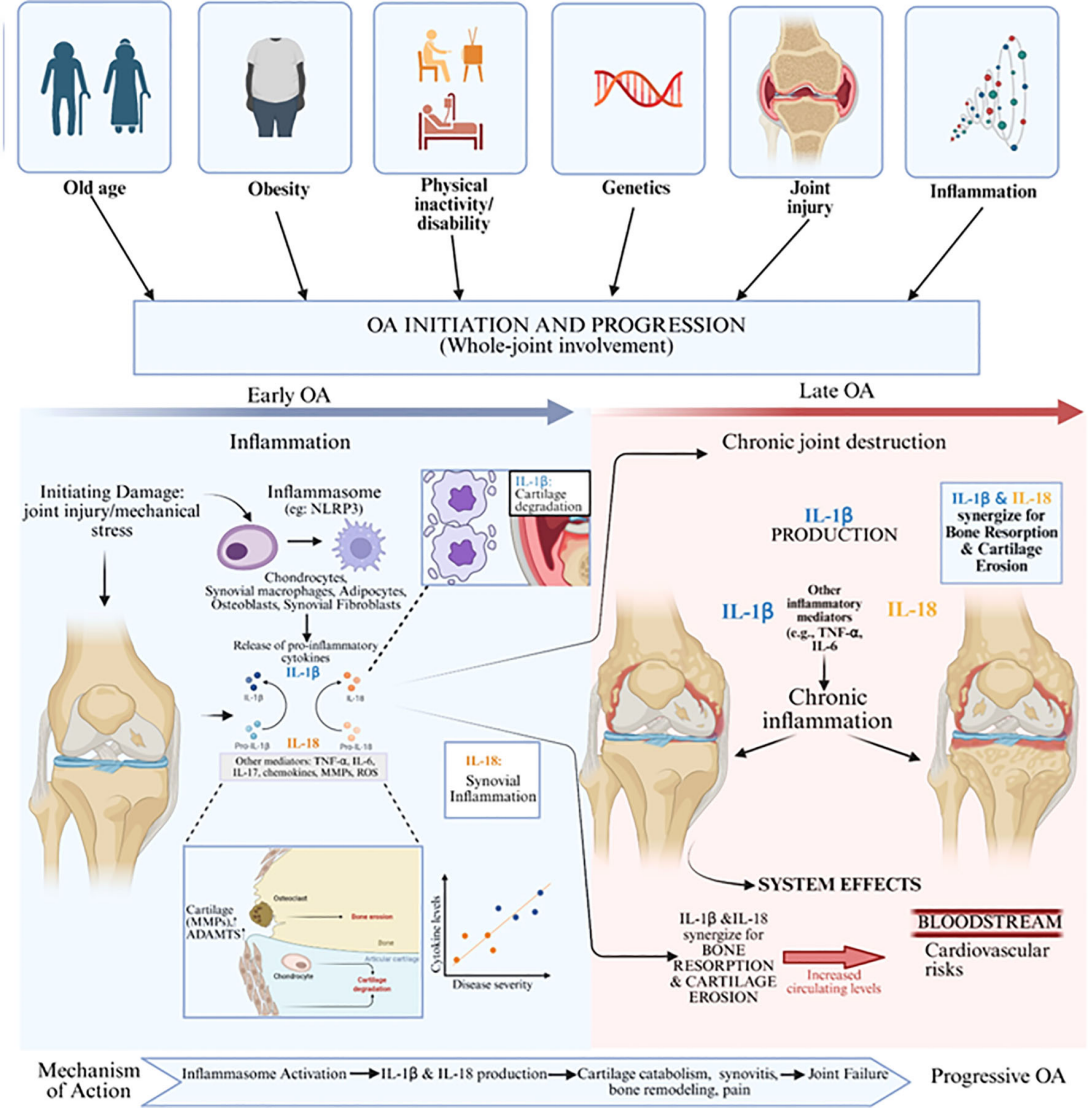
OA progression: IL-1β/IL-18 are activated by inflammasome-mediated signaling in jointtissues. These pro-inflammatory cytokines promote synovial inflammation, cartilage degradation, and joint destruction by inducing the release of MMPs and ADAMTSs. These further triggers chronic inflammation and chondrocyte apoptosis, thereby contributing to structural damage to the joint. Persistent elevation leads to progressive joint failure and disease progression. (Created using Bio-Render, Bio-Render license: XF29WPDLCA).

IL-1β signals through the IL-1 receptor, activating immune cells and amplifying the pro-inflammatory response. IL-1β induces IL-6 production, which elicits osteophyte formation and causes abnormal bone remodeling (8). IL-18 also affects bone by acting through receptors on osteoclasts and osteoblasts (8). Consequently, the IL-1β/IL-18 axis is associated with abnormalities in cartilage and bone, contributing to the structural alterations observed in OA (**Figure 1**). This occurs by indirectly promoting osteoclast activation through elevated RANKL expression on osteoblasts and by impeding cartilage repair (9, 10). The NOD-like receptor protein 3 (NLRP3) inflammasome, a common upstream element, activates these cytokines, often resulting in their simultaneous release under stress, underscoring a crucial pathway in the development of OA and other inflammatory joint disorders (11). Specifically, the proteolytic activation of IL-1β and IL-18 by caspase-1 results from inflammasome activity and subsequently leads to the upregulation of various catabolic factors, including matrix metalloproteinases (MMPs) (12).

Beyond the common inflammasome-dependent maturation, interleukins IL-1β and IL-18 exhibit extensive reciprocal regulation that amplifies inflammatory responses during OA progression. IL-1β-mediated activation of the NF-κB pathway increases transcription of pro-IL-18 and inflammasome components, thereby enhancing IL-18 processing and secretion. IL-18 primarily increases the production of inflammatory mediators, including IL-6, TNF-α, and IFN-γ, thereby further reinforcing NF-κB signaling and sustaining IL-1β expression. This bidirectional cytokine interaction forms a self-perpetuating inflammatory loop that intensifies chondrocyte catabolism, extracellular matrix degradation, oxidative stress, and synovial inflammation, thereby accelerating joint destruction in OA (13, 14).

Subsequently, interleukins (IL-1β and IL-18) activate downstream signaling cascades, specifically NF-κB and MAPK pathways, leading to the expression of comparable target genes, including IL-6, iNOS, and MMPs. Overexpression of IL-1β and IL-18 in osteoarthritic synovial tissue and fluid also promotes the synthesis of reactive oxygen species, thus contributing to oxidative stress and further cartilage deterioration (15). This article highlights the individual and complementary roles of IL-1β and IL-18 in the progression of knee OA, focusing on their shared upstream activation and common downstream signaling pathways. It also provides a comprehensive overview of the interaction between IL-1β and IL-18 in the osteoarthritic landscape. This narrative manuscript was developed after a comprehensive literature search across PubMed and Scopus using the keywords “osteoarthritis”, “interleukins”, “cartilage degradation”, and “immunotherapy”.

## 2 Secretory pathways of IL-1β and IL-18

IL-1β and IL-18 are key drivers of inflammation and contributors to disease progression due to their potent pro-inflammatory properties. The secretion pathways of both cytokines are distinct from those of most secreted proteins, which follow the classical endoplasmic reticulum-Golgi apparatus secretory pathway (15). Both cytokines lack a signal peptide and are released as precursors via a non-classical secretory pathway primarily associated with inflammasome activation and cell death. The distinct yet interconnected mechanisms underlying OA progression are crucial for understanding disease progression and for effectively targeting treatments (15, 16). Both cytokines are mainly produced as biologically inactive precursors, pro-IL-1β and pro-IL-18, which are stored in the cytosol and then cleaved directly by the protease caspase-1, thereby indirectly affecting Ca2+ and calpain-dependent processing of pro-IL-1 (17, 18). In contrast, inflammasome-independent processing of IL-1β has also been demonstrated in caspase-1-deficient mice, which use a different pathway to activate the precursor IL-1β to active IL-1β via neutrophil proteases, including elastase, proteinase-3, granzyme AA, and cathepsin G. Inflammasomes are signaling complexes that are formed or activated by certain cytosolic pattern recognition receptors (PRRs) that act as sensors for pathogen recognition, cellular damage, cellular stress or danger signals, or cellular homeostatic disruption, and nucleate oligomerization of an adaptor protein, ASC (apoptosis-associated speck-like protein containing a CARD), into an inflammasome complex (19). Several inflammasome pathways contribute to OA progression, and the most thoroughly investigated are the NLRP3 inflammasomes (12). Inflammasomes can be further classified into two types, depending on the caspase they activate: canonical or non-canonical inflammasomes. The canonical pathway is the largest group, and the protein sensors required to assemble canonical inflammasomes are AIM2, CARD8, Pyrin, and NLRP1, 3, and 6. These protein sensors recognize pathogens, thereby further driving ASC recruitment—the caspase activation and recruitment domain (CARD) of ASC recruits caspase-1 activation (19). Mohamed Ghait et al. reported that the non-canonical inflammasome activates caspase 4 and 5 in humans. It is a lipid-caspase complex that provides host defense against gram-negative bacteria. Additionally, it has been reported to function without dedicated sensor proteins (20).

**Figure 2:**
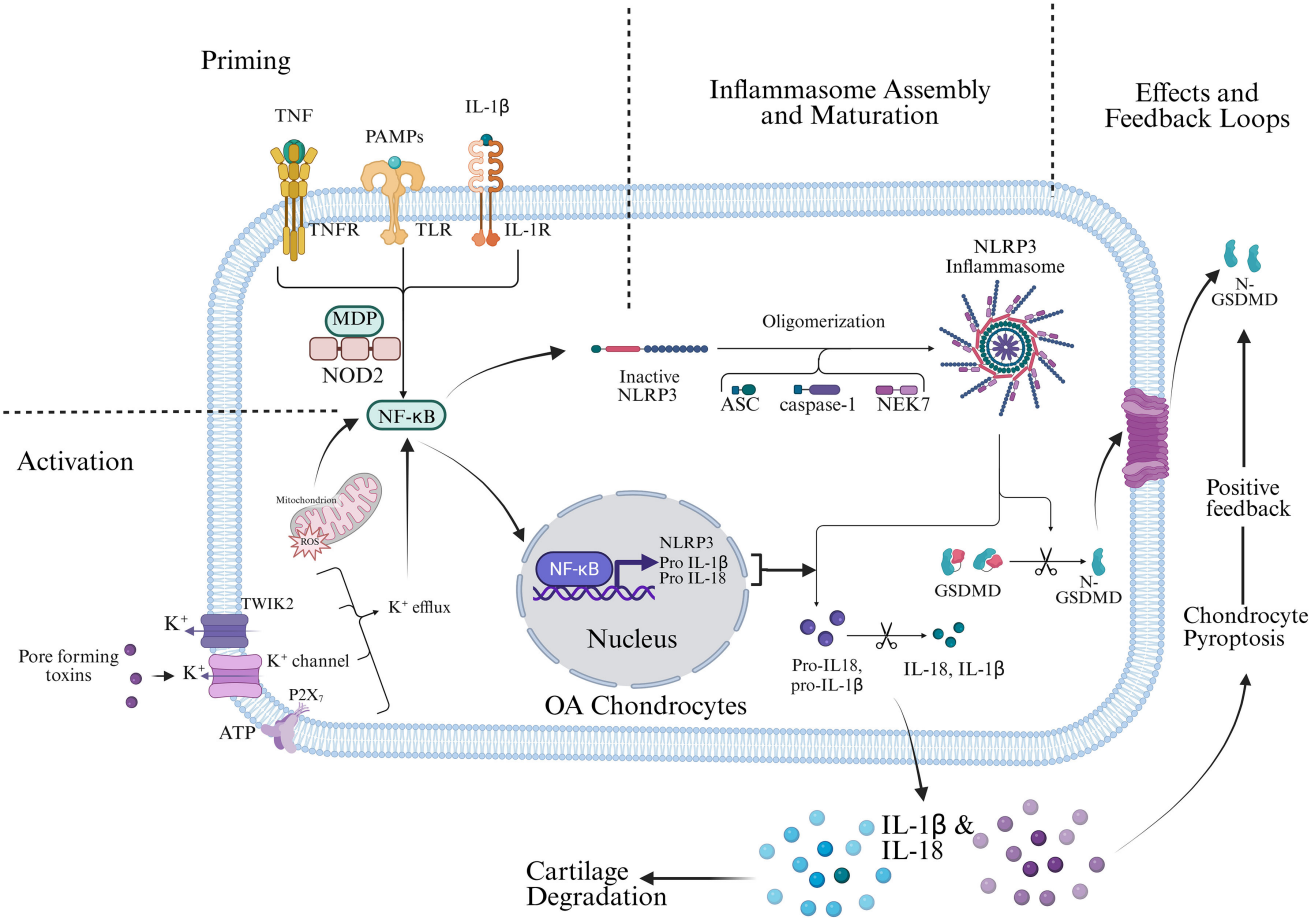
NLRP3 inflammasome activation pathway in OA chondrocytes: The NLRP3 inflammasome is triggered by mitochondrial dysfunction, elevated ROS levels, and ionic imbalance. The NLRP3-ASC-caspase-1 complex drives the activation and maturation of IL-1β and IL-18 from their pro-forms. Once mature, these cytokines further promote inflammatory signaling pathways and cell death. TLR, Toll-like receptor; TNF, tumor necrosis factor; TNFR, tumor necrosis factor receptor; PAMPs, pathogen-associated molecular patterns; IL-1R, interleukin receptor; NLRP3, NLR family pyrin domain-containing 3; NF-κB, nuclear factor kappa B; ASC, apoptosis-associated speck-like protein containing a CARD; NEK7, neverin mitosis gene A-related kinase 7; GSDMD, gasdermin D; TWIK2, tandem of P domains in a weak inwardly rectifying K+ channel 2; P2X7, purinergic receptor P2X ligand-gated ion channel 7. (Created using Bio-Render, Bio-Render license: VG29WPDLQD).

Caspase-1 or caspase-11/4/5, once activated, cleaves pro-cytokines into mature IL-1β and IL-18 and processes gasdermin D (GSDMD), a pore-forming protein. Once the cytokines mature, they cross the plasma membrane to reach the extracellular space, bypassing ER-Golgi-mediated secretion. This route involves GSDMD-mediated pore formation, in which the N-terminal GSDMD oligomerizes at the plasma membrane, forming pores that induce cell rupture and allow IL-1β and IL-18 to diffuse into the extracellular space. This mechanism, called pyroptosis, allows the release of cytokines even from viable cells under certain conditions. Therefore, there is a shared secretory pathway for IL-1β and IL-18 (21–23) (**Figure 2**).

In OA, inflammasomes are triggered in the joints by damage-associated molecular patterns (DAMPs) and pattern recognition receptors (PRRs). Certain hydroxyapatite (HA) crystals, such as calcium crystals found in joints, prompt the release of IL-1β and IL-18. These hydroxyapatite crystals specifically stimulate the secretion of IL-1β and IL-18, a process that is caspase-1-dependent and requires conditions such as potassium efflux, reactive oxygen species (ROS) generation, phagocytosis, and lysosomal protease release, thereby activating NLRP3 (24, 25). Signaling pathways, such as NF-κB activation by TLR4 sensitization in synoviocytes and macrophages, prime pro-IL-1β and pro-IL-18. The release of DAMPs from cartilage degradation under mechanical stress or high ROS levels disrupts the homeostatic state of articular chondrocytes and triggers the release of pro-inflammatory cytokines. Extracellular ATP production enhances inflammasome priming and assembly (26).

Research conducted both *in vivo* and *in vitro* has demonstrated that persistent inflammasome activation leads to increased expression of the pro-inflammatory cytokines IL-1β and IL-18 in the joints. Furthermore, crystal detection in the synovial fluid was associated with elevated levels of IL-1β and IL-18, which were notably higher in samples containing crystals. This finding supports the existence of a crystal-inflammasome-cytokine axis in the pathophysiology of OA (27) (**Table 1**).

**Table 1:** OA specific-upstream triggers, cytokine processing and secretion mechanisms of IL-1β and IL-18 in the pathogenesis of Osteoarthritis.

Mechanistic stage	IL-1β	IL-18	OA-specific upstream triggers / regulatory mechanisms	References
Priming	Transcription of IL-1β is initiated by TLR2/TLR4 and IL-1R dependent signalling, leading to pro-IL-1β production	Constitutively expressed and upregulated through inflammatory stimuli.	DAMPs (HMGB1, fibronectin fragments, S100A8/A9, hyaluronan fragments), mechanical stress, ageing, obesity, synovitis, and NF-κB activation.	(27)
Inflammasome activation	NLRP3 inflammasome mainly activates caspase-1 to further process pro-IL-1β into mature IL-1β.	NLRP3-dependent caspase-1 activation converts pro-IL-18 into mature IL-18.	Mitochondrial ROS, mtDNA release, K^+^ efflux, extracellular ATP, crystal-induced lysosomal damage, and ER stress.	(28)
Cytokine maturation	Proteolytic processing by Caspase-1 generates biologically active IL-1β.	Caspase-1 cleavage that generates biologically active IL-18.	Persistent inflammasome activity in OA chondrocytes, synovial macrophages, and fibroblast-such synoviocytes.	(29)
Non-classical secretion	Secreted through gasdermin D pores, secretory lysosomes, extracellular vesicles, and plasma membrane shedding.	Released by unconventional pathways during pyroptosis.	Gasdermin D pore generation, extracellular vesicle release, and inflammasome-generated membrane permeabilization.	(30)
Release during inflammatory cell death	Mainly released during pyroptosis and passive cell lysis, amplifying local inflammation.	Released generated during pyroptosis, contributing to synovial inflammation and cartilage degeneration.	Pyroptotic chondrocytes, activated synovial macrophages, necrotic cells, and extracellular matrix degradation.	(31)

Dai et al. showed that IL-18 triggers MMP production in chondrocytes, and IL-1β amplifies this effect (31). Another study by Ma et al. reported that plasma IL-1Ra levels are positively associated with radiographic severity in early knee OA via activation of the NLRP3 inflammasome, thereby promoting caspase-1-dependent maturation of IL-1β and IL-18 (32).

Collectively, these highlight that dysregulated secretion of the interleukins IL-1β and IL-18 is a characteristic feature of the OA inflammatory microenvironment, in which sustained inflammasome activation further contributes to cartilage destruction and disease progression (29).

## 3 Inflammasome biology of IL-1β and IL-18

IL-1β and IL-18 are the major pro-inflammatory cytokines that are co-released as cytosolic precursors and require inflammasome activation via secondary proteolytic cleavage in osteoarthritic joints, but are regulated differently in the release mechanism (21). IL-1β is minimally expressed under homeostatic conditions and requires transcriptional induction in blood monocytes, macrophages, and dendritic cells upon activation with toll-like receptor (TLR) ligands and other cytokines such as tumor necrosis factor (33). IL-18, on the other hand, is expressed in blood monocytes, intestinal epithelial cells of healthy humans, murine macrophages, dendritic cells, and endothelial cells. In contrast to IL-1, which starts equation beta, IL-18 is present as a pre-existing cellular pool that requires inflammasome engagement for activation and is poised for rapid release (34). IL-1β required prior transcriptional induction before maturation. Whereas IL-18 is primarily involved in the immediate release following inflammasome activation, this variation in regulation may influence the temporal dynamics of inflammatory responses during OA progression (33).

In OA, NLRP3 inflammasome activation primarily requires a two-step process: priming is mediated by TLR/NF-κB signaling, with lipopolysaccharide (LPS), a TLR4 agonist, as the primary stimulus that induces the expression of NLRP3, pro-IL-1β, and pro-IL-18. Furthermore, LPS also increases IL-18 mRNA expression (35). Other stimuli, such as CpG oligonucleotides (TLR9 agonists) and Sendai virus infection, can contribute to IL-18 upregulation. These subsequent activation triggers inflammasome assembly, caspase-1 activation, and further maturation of IL-1β and IL-18. Since these mechanisms have been extensively reviewed elsewhere (33), this review focuses on their relevance to OA.

Although IL-1β and IL-18 are key mediators in the innate immune system, their crucial functions are to maintain a delicate balance that protects the host from viral and bacterial invaders while preventing tissue damage from excessive inflammation. To achieve this, mammals have likely evolved a two-step regulatory system for cytokines (36). Unlike most secreted proteins, the cytosolic precursors of IL-1 and IL-18 are produced and typically require secondary proteolytic cleavage by the inflammasome for their activation and release. Inflammasomes are multiprotein complexes made up of sensor molecules, such as NLR family proteins (NLRP1, NLRP3, NLRC4, and NLRC5), AIM2, or the cytosolic RNA helicase RIG-I (37). They recruit caspase-1 through the adaptor protein ASC/Pycard to perform enzymatic activity. The assembly of inflammasomes and processing of IL-1β and IL-18 are initiated by various stimuli, including bacteria, viruses, and danger-associated molecular patterns (DAMPs), such as ATP (13). Cross-talk between IL-1β and IL-18 is mediated by common intracellular signaling pathways, including NF-κB, MAPKs (ERK, JNK, and p38), PI3K/Akt, and HIF-2α. Collectively, these signaling pathways enhanced the production of inflammatory mediators, including IL-6, TNF-α, nitric oxide, prostaglandin E_2_, and reactive oxygen species, which contribute to persistent chronic synovitis and cartilage destruction (38).

Furthermore, IL-1β-induced NF-κB activation promotes NLRP3 inflammasome expression, thereby facilitating sustained IL-18 maturation, whereas IL-18 augments IL-1β production through IFN-γ- mediated inflammatory signaling. This reciprocal amplification contributes to persistent low-grade inflammation characteristic of OA (39). Similarly, IL-18 alone exhibits relatively modest catabolic effects, with stronger pathological activity observed in the presence of IL-1β or other cytokines such as TNF-α. These discrepancies may arise from differences in disease stage, patient heterogeneity, obesity, aging, mechanical stress, species-specific variation, and experimental methodologies. Therefore, IL-1β and IL-18 should be viewed as components of a broader inflammatory network rather than isolated drivers of OA progression, highlighting the need for biomarker-guided and combination therapeutic strategies (40). Therefore, IL-1β and IL-18 should be considered complementary components of a coordinated inflammatory network rather than independent mediators, which supports the rationale for biomarker-guided and combination therapeutic approaches in OA management.

## 4 Signaling pathways

In OA conditions, IL-1β binds to IL-1R1 and exerts its effects, whereas IL-18 interacts with the IL-18Rα/β receptor. When a ligand binds to the receptor, it recruits MyD88, which in turn activates IRAKs and TRAF6. This signaling axis further activates several intracellular signaling pathways, such as NF-κB, HIFs, Wnt, and PI3K/Akt.

### 4.1 HIF signaling pathway

Hypoxia-inducible factors (HIFs) are transcription factors that become active in response to low oxygen levels or inflammatory signals to ensure cell survival under these conditions (41). HIF family comprises HIF-1α, HIF-2α (EPAS1), or HIF-3α, which forms a heterodimer with a β-subunit (HIF-β), also known as aryl hydrocarbon receptor nuclear translocator (ARNT) (**Table 2****).**

**Table 2:** Table summarizing roles of IL-1β and IL-18 in different cell types, signaling pathways.

Cell type	Role of IL-1β	Role of IL-18	Impact on OA progression	References
Articular chondrocytes	↑ NF-κB/MAPK activation, ↑ MMP-1/3/13, ↑ ADAMTS-4/5, ↑ COX-2, ↓ Collagen II, ↓ Aggrecan	↑ ROS generation, ↑ apoptosis, ↑ pyroptosis, ↑ ferroptosis, ↑ matrix-degrading enzymes	Accelerates cartilage degeneration and loss of ECM integrity.	(42)
Synovial fibroblasts (FLS)	↑ IL-6, ↑ IL-8, ↑ MCP-1, ↑ PGE_2_, ↑ MMP secretion	↑ Chemokine production, ↑ inflammatory cytokines, ↑ immune-cell recruitment	Sustains chronic synovial inflammation and joint destruction.	(43)
Synovial macrophages	↑ M1 polarization, ↑ TNF-α, ↑ IL-6, ↑ inflammatory amplification	↑ Inflammasome activity, ↑ IFN-γ production, ↑ macrophage activation	Maintains chronic innate immune activation and cytokine amplification.	(44)
Osteoblasts	↑ RANKL expression, ↑ inflammatory mediators, ↓ osteogenic homeostasis	↑ Inflammasome-mediated dysfunction, ↑ abnormal bone remodeling	Contributes to subchondral bone sclerosis and osteophyte formation.	(46)
Osteoclasts	↑ Osteoclast differentiation and bone resorption	↑ Osteoclast activation through inflammatory signaling	Accelerates abnormal subchondral bone remodeling.	(46)
Mesenchymal stem cells (MSCs)	↓ Chondrogenic differentiation, ↓ regenerative potential	↑ Oxidative stress, ↑ inflammasome activation, ↓ cartilage repair capacity	Impairs endogenous cartilage regeneration.	(47)
Endothelial cells	↑ VEGF expression, ↑ angiogenesis	↑ Endothelial activation, ↑ vascular inflammation	Enhances synovial angiogenesis and inflammatory cell infiltration.	(48)
T cells and NK cells	↑ Th17-associated inflammatory responses	↑ IFN-γ secretion, ↑ Th1 and NK cell activation	Amplifies chronic immune responses within the osteoarthritic joint.	(49)

↑ indicates upregulation or increased expression/activity/level.

↓ indicates downregulation or decreased expression/activity/level.

Under normal conditions, HIF-α undergoes oxygen-dependent hydroxylation by prolyl hydroxylases, PHD1, PHD2, and PHD3, with the help of certain cofactors such as oxygen (O_2_), iron (Fe^2+^), alpha-ketoglutarate, and ascorbic acid. After hydroxylation, HIF-α is recognized by the pVHL (von Hippel-Lindau) complex, which promotes polyubiquitination and targeting for degradation, thereby inhibiting transcriptional activation under normal oxygen levels (49). Additionally, Factor Inhibiting HIF (FIH) is another enzyme that inhibits HIF-alpha signaling in normoxic conditions by hydroxylating an asparagine residue (Asn803 in human HIF-1α, Asn851 in human HIF-2α) within the C-terminal transactivation domain (CTAD) of HIF-1α and HIF-2α (50). When oxygen levels are low, the functions of PHDs and FIH are suppressed, resulting in reduced hydroxylation and a buildup of HIF-1α proteins. Accordingly, pVHL-mediated ubiquitination and proteasomal degradation of HIF-1α are inhibited, thereby causing its stabilization and accumulation in the cell cytoplasm. Once accumulated, HIF-1α translocates to the nucleus, where it pairs with HIF-β to form an active transcriptional complex that attaches to hypoxia-responsive elements (HREs) (51).

**Figure 3:**
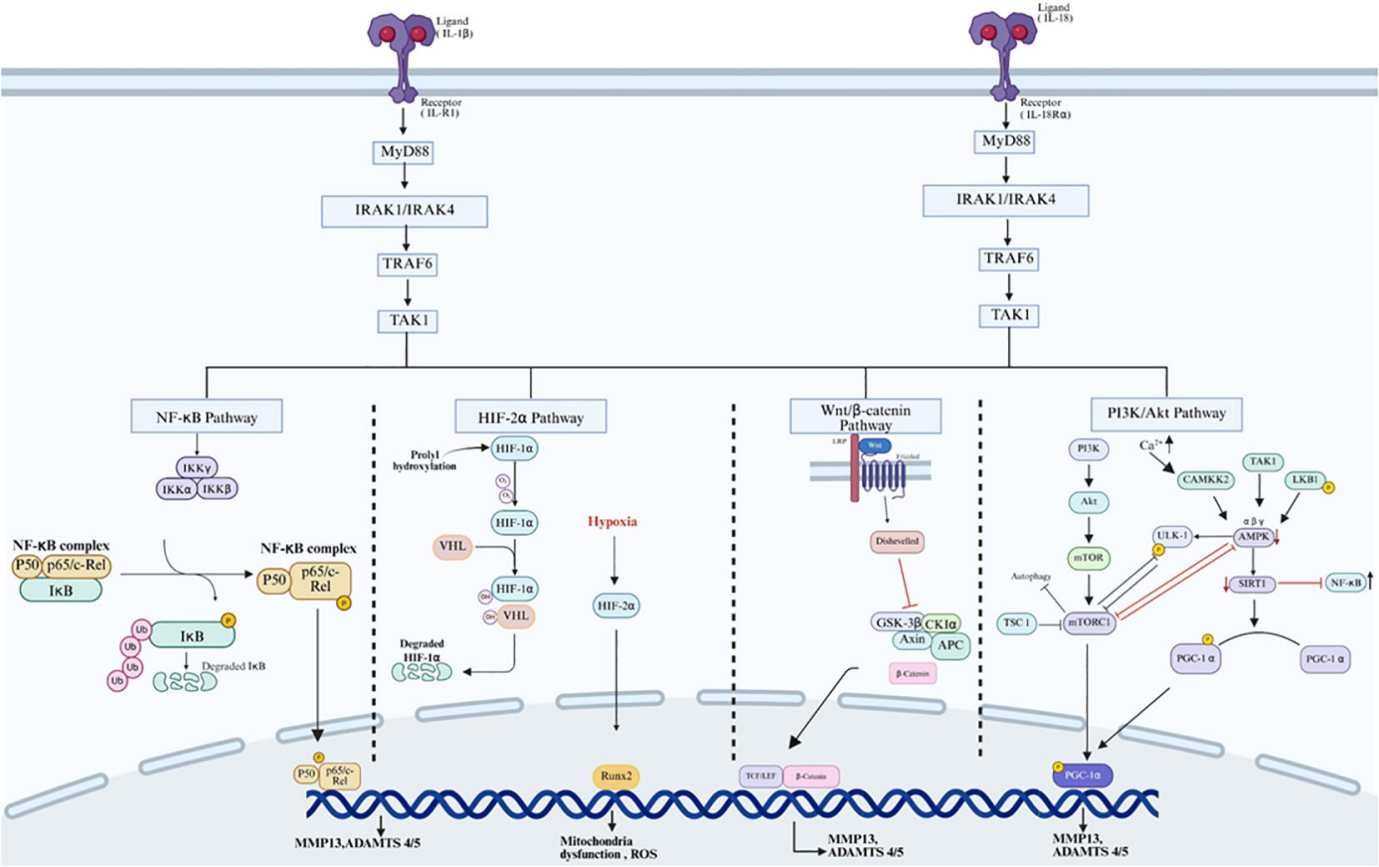
Under hypoxia and inflammatory stimuli, HIF-1α, NF-κB, PI3K/Akt/mTOR, and Wnt signaling pathways release degradative enzymes such as MMPs and ADAMTs. The PI3K/Akt/mTOR signaling cascade activates AMPK, which further upregulates SIRT1 expression, thereby inhibiting mTOR and activating the transcription factor phospho-PGC1α, leading to mitochondrial dysfunction and increased ROS levels during the inflammatory response. HIF-1α, hypoxia-inducible factor-1 alpha; VHL, von Hippel-Lindau tumor suppressor protein; TRAF, tumor necrosis factor receptor-associated factor; NIK, Nuclear factor kappa-light-chain-enhancer of activated B cells-inducing kinase; IKKα, Inhibitory kappa B kinase α; p100, NF-κB precursor protein 100; RelB, v-rel avian reticuloendotheliosis viral oncogene homology B; p52, NF-κB subunit p52; MMPs, Matrix metalloproteinases; ADAMTs, a disintegrin and matrix metalloproteinase with thrombospondin motifs. CaMKK2, Calcium/calmodulin-dependent protein kinase kinase 2; TAK1, Transforming growth factor beta-activated kinase-1; LKB1, Liver kinase B1; AMPK, AMP-activated protein kinase; SIRT1, Sirtuin 1; PGC1 α, peroxisome proliferator-activated receptor gamma coactivator 1-alpha; ULK1, Unc-51 like autophagy activating kinase1; PI1K, Phosphoinositide 3-kinase; AKT, Protein kinase B; mTOR, mechanistic target of rapamycin; TSC, Tuberous sclerosis complex; ROS, Reactive oxygen species; GSK-3β, Glycogen synthase kinase-3 beta; CKIα, Casein kinase 1 alpha; APC, Adenomatous polyposis coli. (Created using Bio-Render, Bio-Render license: LF29WPDMM2).

HIF-1α, in conjunction with HIF-β, acts as a transcriptional factor for a wide range of genes involved in erythropoietin (EPO), glucose transporter-1 (GLUT-1), and vascular endothelial growth factor A (VEGFA). HIF signaling is responsible for these responses as it promotes metabolic reprogramming and supports cellular adaptation to hypoxic stress. *In vivo*, HIF-1α is an important survival factor for chondrocytes in hypoxic environments. In contrast, HIF-2α is associated with endochondral ossification, as it inhibits Col10a1 expression, thereby promoting matrix degradation (52) (**Figure 3**).

Articular cartilage is devoid of vascularization; hence, articular chondrocytes are normally in a hypoxic state (53). HIF-1α and HIF-2α are activated in both mice and humans. HIF-1α acts as a protector of articular cartilage by downregulating MMP13 expression, which induces the OA phenotype in mice. In OA cartilage, the expression of HIF-2α is higher than that in normal cartilage in both mice and humans (54). This overexpression of HIF-2α triggers the destruction of articular cartilage in mice and rabbits. Saito et al. demonstrated that OA development is regulated by HIF-2α expression, suggesting a central role for HIF-2α as a transactivator and therapeutic target for OA (52).

IL-1β upregulates HIF-2α expression via the NF-κB signaling pathway, while increasing ROS levels and destabilizing PHD activity. IL-18 promotes inflammasome activation and induces mitochondrial stress, increasing ROS and triggering HIF regulation. Activation of HIF-2α led to an increase in the expression of Runx2, MMP1, MMP3, MMP13, ADAMTS4, and NOS2, all of which contribute to cartilage degradation. In addition to the hypoxic, inflammatory NF-κB signaling, mTORC1 can indirectly stabilize HIF proteins by elevating ROS levels and modulating PHD activity in articular cartilage (55).

### 4.2 NF-κB signaling pathway

Nuclear Factor kappa-light-chain-enhancer of activated B cells (NF-κB) is a family of transcription factors activated by pro-inflammatory cytokines and plays vital roles in inflammation, differentiation, proliferation, and survival of mammalian cells (56). The mammalian NF-κB subfamily comprises five proteins: RelA or p65, RelB, c-Rel, NF-κB1 or p50 (p105 as an inactive precursor), and NF-κB2 or p52 (p100 as an inactive precursor) (57). All NF-κB proteins share a common N-terminal Rel-homology domain. This domain enables dimerization into homo- and heterodimers, nuclear localization, DNA binding, and interaction with NF-κB inhibitors. The resulting dimers fall into two categories: those containing p50 and/or p52, and those containing c-Rel, p65, and RelB, which possess a transcriptional activation domain (TAD) that drives gene transcription (58). In contrast, p50 and p52 lack a TAD and therefore cannot initiate transcription. In eukaryotes, the most common dimers are p65:p50 and RelB:p52, which activate gene transcription. NF-κB complexes interact with NF-κB inhibitory proteins (IκBs), including IκBα, IκBβ, IκBγ, IκBϵ, IκBζ, and Bcl-3. This interaction blocks NF-κB phosphorylation and activation. Pro-inflammatory cytokines activate the IκB kinases (IKKs), which phosphorylate IκB, leading to its degradation. This frees NF-κB to translocate to the nucleus and bind the promoters of target genes (26) (**Figure 3**).

Articular chondrocytes express receptors for pro-inflammatory cytokines, such as TNF-α or IL-1β, which trigger the activation of the NF-κB signaling pathway, either alone or in crosstalk with bone morphogenetic protein (BMP), Wnt, and other signaling cascades. This crosstalk activates the secretion of several matrix metalloproteinases (MMPs), including MMP1, MMP2, MMP3, MMP7, MMP8, MMP9, MMP13, and ADAMTS4 and ADAMTS5, leading to articular cartilage breakdown (58, 59).

The NF-κB signaling pathway exacerbates OA by inducing PGE2 (Prostaglandin E2), nitric oxide (NO), and COX2 (cyclooxygenase-2), thereby promoting articular cartilage damage through inflammation, the release of catabolic factors, and chondrocyte apoptosis. NF-κB also upregulates HIF-2α, E74-like factor 3 (ELF3), and RUNX2, which in turn trigger expression of the ADAMTS5 and MMP13 proteases, directly promoting the differentiation of pre-hypertrophic articular chondrocytes into terminally differentiated chondrocytes (60). NF-κB also interacts with mTOR (via AKT) to inhibit autophagy. Chronic NF-κB activation stabilizes senescence-associated secretory phenotype (SASP) and sensitizes cells to regulated necrosis by altering the balance between pro-survival and pro-death signals (26).

A study by Fan et al. demonstrated the role of NF-κB using inhibitors, which significantly blocked MMP induction and collagen-II repression in normal human articular chondrocytes (60).

### 4.3 Wnt signaling pathway

Wingless-Integration site-1 (Wnt) glycoproteins comprise poorly soluble signaling proteins that regulate chondrogenic differentiation, chondrocyte hypertrophy, and growth plate chondrocytes (61). Based on their dependence on β-catenin, Wnt pathways are classified into two main types: canonical and non-canonical. The canonical pathway is mainly dependent on β-catenin, whereas the non-canonical pathway is independent of β-catenin signaling. β-catenin binds to cadherin, a subunit of the cadherin protein complex, and forms a complex with GSK3β after phosphorylation (26).

The canonical Wnt signaling pathway is activated by binding of Wnt ligand to frizzled and the co-receptor LRP5/6. This interaction inhibits proteasomal degradation, stabilizing β-catenin in the cytoplasm, which then translocates to the nucleus and activates the transcription of Wnt target genes. A balanced optimal level of Wnt canonical signaling is essential for maintaining healthy articular cartilage. Suppressing β-catenin to inactivate the canonical Wnt pathway further can be achieved by overexpression of the inhibitor of β-catenin and T-cell factor (ICAT) in Col2a1-ICAT transgenic mice, which causes chondrocyte apoptosis. Wnt signaling reduces the planar cell polarity (PCP)/JNK-mTORC1-PTHRP cascade, thereby reducing catabolic activity (62).

In patients with OA, β-catenin is upregulated, leading to chondrocyte hypertrophy, reduced matrix synthesis, cartilage degeneration, and osteophyte formation. Wnt signaling also affects subchondral bone remodeling and determines the fate of cell progenitors. Irregular Wnt signaling can reduce autophagy and increase cellular senescence in progenitors, thereby limiting their regenerative potential (63) (**Figure 3**).

Sarrion et al. reported that Wnt activation can significantly downregulate anabolic markers (Col2A1 and ACAN) in human cartilage explants under mechanical loading, indicating that Wnt activation disrupts chondrocyte homeostasis and contributes to early OA (64).

### 4.4 PI3K/Akt/mTOR signaling pathway

Inflammatory cytokines and other molecules can initiate the PI3K/Akt/mTOR signaling pathway by activating receptor tyrosine kinases (RTKs) and G protein-coupled receptors (GPCRs), ultimately activating PI3K. PI3K is composed of three classes of molecules: class I, class II, and class III. Pi3K is composed of two subunits: the catalytic (p110s) and regulatory subunit (p85), and the binding between these two stabilizes PI3K. Stabilized PI3K exposes the site where RTKs and GPCRs activate it. Once PI3K is activated, it converts phosphatidylinositol-4,5-bisphosphate (PIP2) into phosphatidylinositol-3,4,5-triphosphate (PIP3), which further activates AKT, which acts as a messenger in PI3K signaling. This activation is regulated by phosphatase and tensin homolog (PTEN), which reverses PIP3 to PIP2 (65). Further, activation of PI3K generates PIP3, leading to AKT phosphorylation and subsequent activation of mTORC1, while PTEN negatively regulates this pathway (26) (**Figure 3**).

Activated AKT can directly stimulate mTORC1 at Ser 2448 and indirectly by phosphorylating tuberous sclerosis complex 2(TSC2). The tuberous sclerosis complex (TSC) comprises two subunits, TSC1 and TSC2, which function as negative regulators of mTOR. TSC1 and TSC2 functions can be inhibited by AKT-mediated TSC2 inactivation, thereby activating mTORC1. The activation of mTORC1 is enhanced downstream by the conversion of Rheb-GTP to Rheb-GDP. IL-1β activates PI3K/Akt but mainly favors mTOR activation and autophagy suppression, whereas IL-18, activated by high ROS levels, indirectly activates mTOR. mTOR activation inhibits unc-51-like kinase (ULK1), further inhibiting autophagy, promoting anabolic activity, and causing mitophagy (66).

Sustained mTOR activation further promotes chondrocyte senescence, suppresses autophagy, and enhances the senescence-associated secretory phenotype (SASP), thereby reinforcing chronic inflammation and cartilage degeneration in OA. Pharmacological mTOR inhibition (rapamycin) restores autophagy, reduces ROS, and attenuates IL-1β–induced senescence in cartilage models (experimental support exists showing rapamycin mitigates IL-1β effects and rescues chondrogenic capacity (45).

A study by Qian et al. suggested that allicin suppresses IL-1β-stimulated activation of the PI3K/AKT pathway, helping to prevent aberrant bone formation and reduce cartilage degeneration in OA mice (67). Another study by Lu et al. suggested that inhibiting the AKT/mTOR signaling pathway can decrease ROS levels and protect articular chondrocytes from IL-1β-induced damage (68).

## 5 Pathological implications of IL-1β and IL-18 in OA context

The IL-1β- and IL-18-mediated signaling enhanced OA progression by driving cartilage catabolism, oxidative stress, cellular senescence, impaired autophagy, and progressive extracellular matrix degradation.

### 5.1 Senescence

Cellular senescence is a hallmark of OA progression and is exacerbated by chronic exposure to IL-1β and IL-18, which in turn promote oxidative stress and persistent inflammatory signaling pathways. Excessive ROS progression activates the p53/p21 and p16INK4A pathways, which further lead to irreversible chondrocyte growth arrest and acquisition of the senescence-associated secretory phenotype (SASP) (69). Senescent cells have increased levels of the lysosomal enzyme β-galactosidase, detected at pH 6, referred to as senescence-associated (SA) β-galactosidase. Cellular senescence can be detected by markers such as senescence-associated (SA) β-galactosidase (SA-βgal), SA heterochromatin, increased p53, p21, and p16, and reduced Wnt2 expression. Chondrocyte senescence is characterized by a decline in the proliferative and anabolic activities of chondrocytes, thereby contributing to OA progression by limiting chondrocyte repair capacity and increasing senescent activity (70–72).

### 5.2 Autophagy

Autophagy is a protective intracellular process that safeguards cells and maintains chondrocyte survival and cartilage integrity (73). During early OA, chondrocytes attempt to maintain their homeostatic state by increasing cell proliferation, which is accompanied by increased levels of collagen types I and II. In early OA, as inflammation increases, ROS levels increase, and oxidative stress alters mitochondrial function. In response, mitophagy is triggered to eliminate malfunctioning mitochondria and safeguard cells. Additionally, this response activates Unc-51-like autophagy activating kinase 1 (ULK1), beclin1, and microtubule-associated protein 1A/1B-light chain 3 (LC3), which initiates autophagosome formation. Autophagy is essential for maintaining chondrocyte homeostasis by addressing the initial stress conditions. Autophagy helps remove damaged organelles and cartilage debris, thereby preserving chondrocyte function (66). During the advanced phase of OA, factors such as mechanical stress, oxidative stress, and inflammatory signals become more pronounced, resulting in chondrocyte apoptosis and reduced cartilage repair capacity. In the initial stages of OA, autophagy serves as a defense mechanism; however, as the disease progresses, its protective role weakens due to ongoing damage. An overactive autophagy process can lead to cellular dysfunction, causing the release of specific factors, such as apoptosis-inducing factor (AIF) and serine protease Omi/high-temperature requirement protein A2 (HtRA2), from compromised mitochondria. This causes an overlap between autophagy and apoptosis, resulting in caspase-independent apoptosis. Hence, in severe OA, a combination of apoptosis and autophagy leads to cell death (74).

In chondrocytes, IL-1β leads to a decline in autophagic flux, characterized by reduced LC-II formation and increased p62 accumulation, thereby triggering increased ECM degradation. IL-18 sustains inflammation, which further dysregulates autophagy, contributing to chondrocyte dysfunction and cartilage degeneration (75).

### 5.3 Hypoxia

Articular cartilage exists in hypoxic conditions in which HIF is stabilized post-transcriptionally by hydroxylation of specific amino acid residues. However, in the presence of molecular oxygen, HIF is degraded via hydroxylation of specific proline residues, leading to pVHL-mediated proteasomal degradation (76). In OA, persistent hypoxia and chronic inflammation, in which both IL-1β and IL-18 participate and promote the destruction of articular cartilage. Under homeostatic conditions, HIF-1α promotes anabolic activity, maintains extracellular matrix synthesis, and cell survival (77). In contrast, under OA conditions, inflammatory cytokines are elevated, thereby maintaining HIF-2α signaling and altering the balance between HIF-1α and HIF-2α signaling (49).

IL-1β activates the HIF signaling pathway via NF-κB and PI3K/Akt in cells such as chondrocytes and synoviocytes. IL-1β-driven HIF-2α activation shifts chondrocytes toward hypertrophic differentiation, thereby causing cartilage degradation via the release of degradative enzymes, as outlined in earlier sections. Jeongki Kim et al. reported that hypoxia induces IL-18 transcription and secretion, thereby activating the HIF signaling pathway (78). This activation occurs through the GTPase Rac1, which is associated with the IL-18 receptor β (IL-18Rβ) subunit via a PI3K-AKT-NF-κB–dependent pathway. In addition, low oxygen levels and high ROS levels promote NLRP3 inflammasome assembly in joint tissues. HIF-1α drives NLRP3 inflammasome activation, triggering further degradation. Hypoxia activates NLRP3, which in turn promotes IL-1β/IL-18 production, thereby sustaining hypoxic signaling and inflammasome priming. Dysregulated hypoxic signaling contributes to oxidative stress and mitochondrial dysfunction. In short, under hypoxia, both IL-1β and IL-18 remodel the behavior of chondrocytes and synovial cells (79, 80).

### 5.4 Cell death

IL-1β and IL-18 promote multiple forms of programmed cell death, such as apoptosis, pyroptosis, necroptosis, and ferroptosis, thereby contributing to progressive cartilage degeneration in OA (81). Recent studies on OA have focused on signaling pathways and target modulation. IL-1β stimulates the expression of TNF, Fas-associated death region protein, and Caspases-3 and -8, hence increasing chondrocyte apoptosis. IL-1β engages death receptor and mitochondrial pathways that drive chondrocyte apoptosis (82). Bao et al. showed that IL-18 treatment in rat chondrocytes increased pro-apoptotic markers and decreased cartilage matrix genes. In this study, they observed downregulation of Collagen II, Sox9, and aggrecan in response to IL-18 stimulation. IL-18 also promotes chondrocyte apoptosis in OA by activating the mTOR pathway (83).

Necroptosis is a caspase-independent form of programmed cell death mediated by signaling pathways such as RIPK1/RIPK3/MLKL under pathological conditions (84). In OA, inflammatory mediators, including IL-1β and TNF-α, promote necroptotic signaling, contributing to chondrocyte loss, synovial inflammation, and cartilage degeneration (85).

Pyroptosis is a form of NLRP3/ASC inflammasome-activated cell death. In OA, caspase-1 is activated by the NLRP3 inflammasome in chondrocytes, fibroblasts, and macrophages, and then cleaves GSDMD, creating pores in the membrane. Pore formation triggers the release of IL-1β and IL-18, amplifying the inflammatory response and causing cartilage degradation (86).

## 6 Impact of IL-1β and IL-18 in OA resident cells

### 6.1 Chondrocytes

IL-1β and IL-18 regulate chondrocyte differentiation and function in OA. IL-1β elevates its own production via autocrine signaling that activates caspase-1 (87). It acts on neighboring cells, such as chondrocytes and synovial macrophages, and activates the degradation of the extracellular matrix, which comprises type II collagen and aggrecan. IL-1β activates signaling pathways such as NF-κB and MAPK (p38/JNK), which upregulate catabolic enzymes such as MMPs (MMP-13) and ADAMTS, and promote stress responses such as iNOS and COX-2 expression. These catabolic enzymes reduce proteoglycan production, thereby causing chondrocyte hypertrophy and dedifferentiation in the upper layer of the articular cartilage. IL-1β drives catabolic effects by promoting chondrocyte apoptosis and reducing the levels of inhibitors, such as tissue inhibitor of matrix metalloproteinases 1 (TIMP1). Recent studies have shown that hypertrophic chondrocytes transdifferentiate into osteoblast-like cells in response to elevated VEGF and RUNX2 expression in OA chondrocytes, leading to osteophyte formation. IL-1β activates signaling pathways such as NF-κB and MAPK (ERK, p38, JNK), which in turn facilitate crosstalk among IL-1β, WNT, and Notch1 signaling (39, 72).

**Figure 4:**
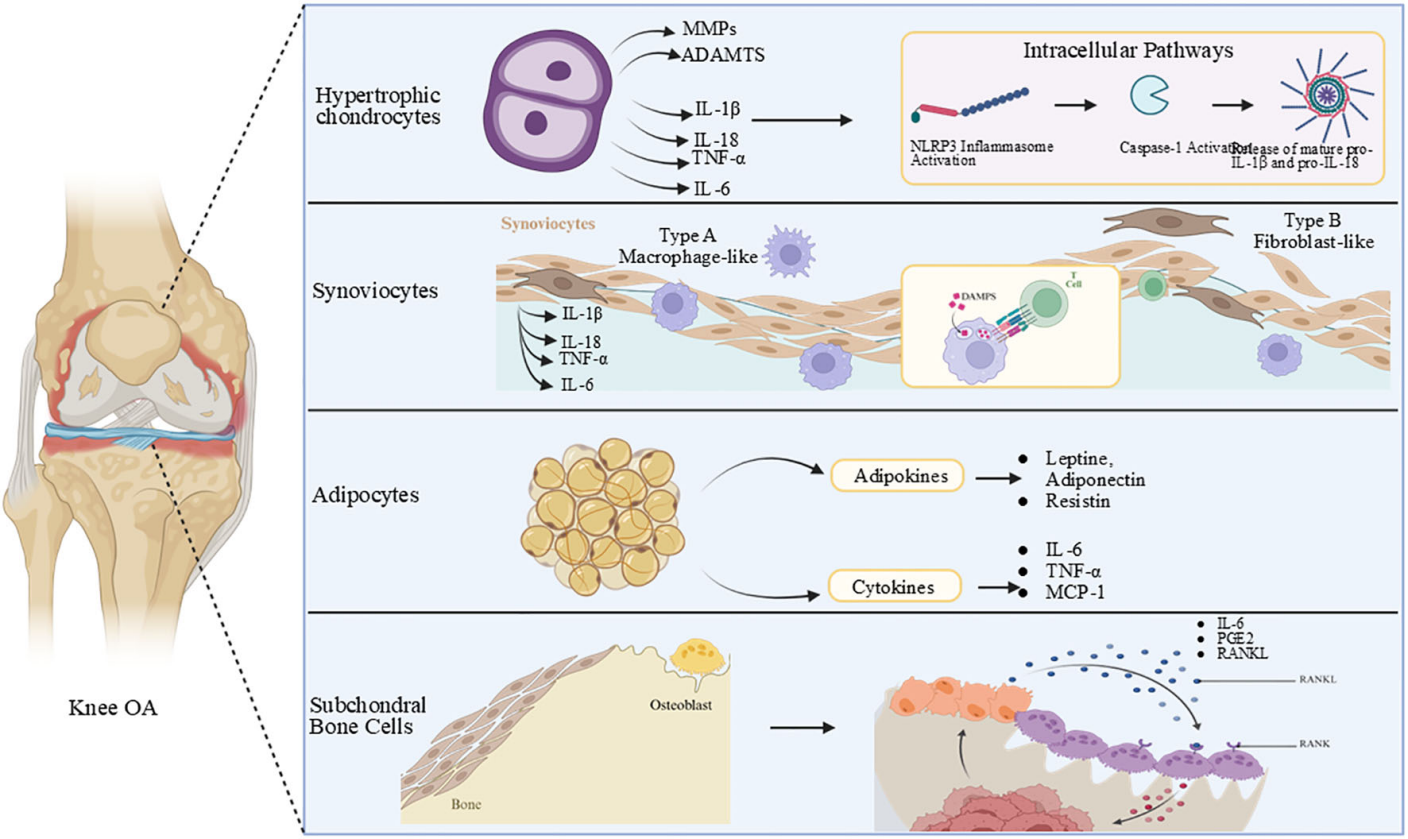
IL-1β and IL-18 in the osteoarthritic joint microenvironment. The illustration depicts resident cells involved in OA pathogenesis that produce pro-inflammatory mediators such as IL-1β, IL-18, TNF-α, and IL-6, thereby promoting inflammation, extracellular matrix degradation, cartilage destruction, and abnormal bone remodeling. MMPs, matrix metalloproteinases; ADAMTS, A Disintegrin and Metalloproteinase with Thrombospondin motifs; IL-1β, Interleukin-1 beta; IL-18, Interleukin-18; TNF-α, Tumor Necrosis Factor-alpha; IL-6, Interleukin-6; NLRP3, NLR family pyrin domain-containing 3; MCP-1, Monocyte Chemoattractant Protein-1; PGE2, Prostaglandin E2; RANKL, Receptor Activator of Nuclear Factor Kappa-B Ligand; RANK, Receptor Activator of Nuclear Factor Kappa-B. (Created using Bio-Render, Bio-Render license: VM29WPDMR8).

IL-18 enhances the production of inflammatory factors that affect synoviocytes and chondrocytes. Zhaozong Fu et al.’s study showed that IL-18 stimulates COX-2 and TNF-α expression and elevates PGE_2_ in primary synoviocytes. IL-18 is produced by chondrocytes in the joint tissue, where it activates the NF-κB cascade, promoting catabolic activity and elevating MMPs (MMP-1/3/13) (6). The release of certain degradative enzymes triggers chondrocyte apoptosis and mitochondrial stress. This leads to high ROS generation, which is further bound to IL-1β/TNF, promoting chondrocyte apoptosis (88) (**Figure 4****).**

### 6.2 Macrophages

Synovial macrophages release pro-inflammatory cytokines, including members of the interleukin-1 (IL-1) family, tumor necrosis factor (TNF), matrix-degrading enzymes, and alarmins. Synovial macrophages are also associated with osteophyte formation in OA. In addition, it was found to enhance pain and joint stiffness in the synovial fluid. Pro-inflammatory macrophages are mostly derived from blood monocytes (89).

IL-1β acts autocrinally or paracrinally in macrophages, maintaining and triggering NF-κB activation, which subsequently promotes macrophage polarization towards a pro-inflammatory state. It also promotes the production of NO and ROS, thereby increasing cellular stress and facilitating crosstalk between macrophages and other cells, such as chondrocytes and FLS, via exosomal and cytokine networks (90). These interactions mainly amplify local inflammation, extracellular matrix degradation, and NLRP3-mediated inflammasome activation, thereby increasing the maturation and release of interleukins such as IL-1β and IL-18 in OA joints (90).

Culemann et al. demonstrated that synovial tissue macrophages are heterogeneous and can be classified into CX3C motif chemokine receptor 1 CX3CR1+ cells and CX3CR1- interstitial macrophages (91). CX3CR1+ lining macrophages mainly form a protective barrier that limits immune cell infiltration and preserves synovial homeostasis. The authors further demonstrated that CX3CR1+ macrophages harbor immunomodulatory markers indicative of an anti-inflammatory phenotype, specifically an M2-like profile, including receptors such as Trem2 and Axl (92). However, disruption of this protective barrier in OA enhanced infiltration of inflammatory macrophages, thereby increasing NLRP3 inflammasome activation and IL-1β/IL-18 release, thereby sustaining synovial inflammation and cartilage degeneration (43).

Elevated levels of IL-18 bind to the IL-18 receptor (IL-18R α/β), activating synovial macrophages. This activation triggers the release of the NF-κB signaling pathway (92). IL-18 promotes the production of other inflammatory signals, such as COX-2 and PGE2. In OA, IL-18 amplifies ROS and maintains an inflammatory environment, thereby amplifying damage to joint tissues (92).

### 6.3 Synoviocytes (fibroblast-like synoviocytes)

Fibroblast-like synoviocytes (FLS) are generally the specialized mesenchymal cells that constitute the synovial lining and play an important role in maintaining joint morphology and homeostasis. During OA, FLS acquire an activated phenotype characterized by increased proliferation and simultaneous production of inflammatory mediators (93). IL-1β is one of the most potent stimulators involved in synoviocyte activation. After binding to IL-1 receptor type 1 (IL-1R1), IL-1β activates the NF-κB and MAPK signaling pathways, which in turn increase the expression of cytokines such as IL-6, IL-8, and TNF-α, as well as chemokines that recruit immune cells into the synovium (94). IL-1β also induces the systemic production of matrix-degrading enzymes, which contribute to extracellular matrix degradation and cartilage destruction.

IL-18 primarily contributes to synovial inflammation by activating synoviocytes via IL-18 receptors and activating the NF-κB and MAPK signaling pathways (94). This further enhanced the production of pro-inflammatory mediators and chemokines, thereby promoting inflammatory responses. IL-18 also acts synergistically with IL-1β to enhance the expression of catabolic factors, which are involved in the degradation of cartilage. Activated fibroblasts, such as the synoviocytes (FLS), drive synovial hyperplasia, angiogenesis, and persistent inflammation through extensive crosstalk with chondrocytes, macrophages, and other joint cells. Consequently, synoviocytes serve as key effectors of the IL-1β/IL-18 axis and represent promising therapeutic targets in OA (95).

### 6.4 Osteoblasts

Osteoblasts are essential regulators of subchondral bone formation, mineralization, and remodeling, which maintain skeletal integrity under physiological conditions. During OA, osteoblast function becomes profoundly dysregulated due to the persistent influence of proinflammatory cytokines (IL-1β and IL-18) (44). These cytokines further disrupt the balance between bone formation and resorption, contributing to pathological alterations in the subchondral bone architecture.

OA subchondral bone studies indicate that IL-1β contributes to osteoblast dysfunction by altering osteogenic activity and promoting a pro-inflammatory phenotype. In osteoarthritic osteoblasts, IL-1β has been associated with impaired matrix production, abnormal expression of bone-remodeling mediators, and disruption of signaling pathways that maintain subchondral bone homeostasis (96). However, the precise effects of IL-1β on osteoblast differentiation may vary according to disease stage and the local joint microenvironment. IL-1β plays a major role in inhibiting osteoblast differentiation and maturation. It suppresses key osteogenic transcription factors, including runt-related transcription factor 2 (Runx2) and osterix/SP7, which are mainly involved in osteoblast lineage commitment and function (96). This suppression further reduces the expression of essential bone matrix proteins, such as osteocalcin and alkaline phosphatase (ALP), ultimately impairing matrix synthesis and mineralization. As a result, osteoblasts in OA pathogenesis exhibit an abnormal phenotype characterized by reduced anabolic activity and defective bone formation.

Additionally, IL-1β- mediated inhibition of osteoblast differentiation significantly alters the osteoblast-mediated regulation of osteoclastogenesis. It upregulates the receptor activator of nuclear factor of kappa-light-chain-enhancer of activated B cells (RANKL) while reducing osteoprotegerin (OPG) levels, thereby shifting the RANKL/OPG ratio to promote osteoclast differentiation and activation, ultimately leading to increased bone reabsorption (97). In OA pathogenesis, osteoblasts fail to maintain normal bone formation and simultaneously promote osteoclast activity, resulting in an imbalance in subchondral bone remodeling.

IL-18 further exacerbates osteoblast dysfunction by activating key inflammatory pathways, including the NF-κB and MAPK cascades (98). These pathway activations further increase the production of proinflammatory mediators, chemokines, and matrix-degrading enzymes, such as MMPs. This proinflammatory osteoblast phenotype increases local joint inflammation and promotes the degradation of both bone and cartilage matrices. Moreover, IL-18 signaling may enhance oxidative stress and further reduce osteoblast viability and function, reinforcing chronic inflammatory states (5).

Together, IL-1β and IL-18 disturb osteoblast function, leading to abnormal subchondral bone remodeling characterized by increased bone turnover, disorganized matrix deposition, and altered mineralization patterns. These changes contribute to subchondral bone sclerosis, a key feature of OA, thereby increasing bone stiffness and reducing shock-absorbing capacity (99). This mechanical imbalance places additional stress on the articular cartilage, hastening its deterioration and disease progression.

In OA, osteoblasts interact abnormally with osteoclasts, chondrocytes, and synovial fibroblasts, perpetuating a vicious cycle of inflammation and tissue destruction. Dysregulated signaling networks involving IL-1β and IL-18 highlight the importance of osteoblasts as active contributors to OA pathogenesis rather than merely passive participants in bone remodeling (24).

### 6.5 Osteoclasts

In OA, osteoclast-mediated bone resorption is primarily driven by proinflammatory cytokines such as IL-1β and IL-18. IL-1β promotes osteoclast differentiation and activation by upregulating RANKL expression and enhancing osteoclast responsiveness to RANK-mediated signaling. This increases cathepsin K and MMP production, leading to bone matrix degradation. IL-18 indirectly promotes osteoclastogenesis by modulating the immune response via T-cell activation and increased secretion of pro-osteoclastogenic cytokines (100). Overactive osteoclasts may cause bone erosion, microfractures, and impaired structural integrity of the articular cartilage. This imbalance between bone formation and resorption creates a self-perpetuating pathological feedback loop that leads to aggressive joint degeneration in OA (101).

Excessive osteoclast activity contributes to subchondral bone thinning and mechanical instability, further increasing stress on the overlying cartilage region. Meanwhile, an enhanced RANKL/OPF ratio not only increases osteoclast maturation but also sustains a proinflammatory microenvironment within the joints (102). Persistent osteoclast activation leads to abnormal bone remodeling, characterized by irregular resorption pits and altered trabecular architecture. Cytokine-driven increases in osteoclast expression are closely linked to cartilage breakdown, and mechanical support may be lost, leading to microdamage. Additionally, osteoclast-derived signaling molecules may further stimulate synovial inflammation, creating a feedback loop that accelerates OA progression (101). Targeting IL-1β and IL-18–mediated osteoclast activation may represent a promising therapeutic strategy for restoring bone-cartilage balance in OA.

### 6.6 Adipocytes/infrapatellar fat pad and adipokines

The IL-1β and IL-18 promote the transition of infrapatellar fat pad (IPFP) adipocytes to a pro-inflammatory phenotype. IL-1β stimulates the production of adipokines, such as leptin and resistin, by IPFP adipocytes, which contribute to synovial inflammation, cartilage degradation, and metabolic dysregulation within the OA joint (103). Furthermore, IL-18 amplifies inflammatory response effects by increasing cytokine release and facilitating immune cell recruitment. Also, IL-1β and IL-18 establish a persistent inflammatory environment in the IPFP, thereby exacerbating inflammation-associated metabolic dysfunction in the OA synovium. Adipokines generated by the infrapatellar fat pad can stimulate IL-1β production, thereby sustaining chronic inflammation and linking it to metabolic dysfunction in OA progression. The IPFP is a local reservoir of inflammatory mediators that may influence multiple tissues and joints, including cartilage, synovium, and subchondral bone. Various adiponectins, such as leptin, adiponectin, and resistin, can modulate chondrocyte metabolism, promoting the expression of catabolic enzymes and inhibiting matrix synthesis (104).

Leptin synergizes with IL-1β and IL-18 to induce MMP production, accelerating cartilage breakdown. IPFP-derived adipokines also stimulate synovial fibroblasts and macrophages, increasing the levels of proinflammatory cytokines and perpetuating joint inflammation. In OA, the IPFP shows increased immune cell infiltration, including T cells and macrophages, which further polarizes adipocytes toward a proinflammatory state. IL-18 mediated signaling (105). In adipocytes, upregulation of chemokines that recruit additional immune cells creates a feed-forward loop of inflammation. The interaction between IPFP adipocytes and osteoblasts or osteoclasts can disrupt subchondral bone remodeling, contributing to bone sclerosis and microstructural changes. Metabolic stress and hypoxia within the IPFP may exacerbate adipocyte dysfunction and enhance pro-inflammatory adipokine secretion (106).

Adipokines also influence angiogenesis within the IPFP and synovium, promoting vascular remodeling, which supports inflammation and tissue degradation. Chronic IPFP inflammation is mainly associated with increased oxidative stress, which further impairs joint tissue homeostasis. Mechanical stress on the IPFP, especially in obese patients, can increase the release of adipokines and cytokines, which are linked to inflammatory pathways (107). The combination of metabolic, inflammatory, and mechanical factors positions the IPFP as an active contributor to the OA pathophysiology. Targeting IPFP-derived adipokines or modulating IL-1β and IL-18 signaling in adipocytes may provide novel therapeutic strategies to reduce joint inflammation and cartilage degeneration (104).

### 6.7 T cells (CD4^+^, CD8^+^, Th subsets)

T cells are central mediators of the immune response in OA joints. IL-1β facilitates the differentiation of naive CD4^+^ T cells into Th17 cells, which produce IL-17 and lead to cartilage degeneration, synovial inflammation, and osteoclast activation. IL-18 drives Th1 polarization and increases interferon-γ (IFN-γ) production by both CD4^+^ and CD8^+^ T cells, thereby intensifying cell-mediated inflammatory responses. IL-1β and IL-18 impair Treg function and stability, thereby enhancing T cell expression and RANKL levels. IL-1β and IL-18 act in concert to promote OA by disrupting immune homeostasis and driving persistent inflammation (108).

Activated T cells can directly interact with osteoclast precursors, enhancing osteoclastogenesis and subchondral bone resorption. The imbalance between proinflammatory cells, such as Th1/Th17 and regulatory T cells, contributes to a self-perpetuating inflammatory loop within the joint. CD8^+^ T cells in OA joints may also produce cytotoxic mediators that induce chondrocyte apoptosis and exacerbate cartilage damage (109). IL-18-driven IFN-γ increases macrophage activation, which further sustains the inflammatory milieu. Chronic T-cell activation in OA promotes synovial hyperplasia, enhances vascularization, and recruits additional immune cells (98). Dysregulated T cell responses are linked to altered cytokine networks, amplifying cartilage and subchondral bone pathology. T cell-derived cytokines contribute to the disruption of the osteoblast–osteoclast balance, further promoting bone remodeling defects. Thus, focusing on Th17/Th1 polarization or enhancing Treg function could be a therapeutic approach to alleviate joint inflammation and slow OA progression (110). Although changes in Th1/Th17 and Treg cell populations have been reported in OA, several proposed mechanistic studies on IL-1β- and IL-18-mediated T-cell regulation are derived from broader inflammatory disease models rather than OA-specific studies. So, the precise mechanisms of action of T-cell subsets in OA progression remain incompletely understood and require further investigation.

### 6.8 Natural killer cells

Natural killer (NK) cells are central mediators of the innate immune system and are increasingly recognized for their role in OA-associated inflammation. IL-18 potently activates NK cells, enhancing their cytotoxic activity and stimulating interferon-γ production, thereby amplifying local inflammatory responses at the joint site. IL-1β indirectly promotes NK cell recruitment by inducing chemokine production in synovial and stromal cells (111). After activation, NK cells interact with macrophages and other immune cells, driving further cytokine release and inflammation. The significant role of NK cells in OA remains underexplored. Significantly, IL-1β-mediated inflammatory signal enhances IL-18-driven NK cell activation in the OA synovium, contributing to OA progression. These cells are believed to contribute to tissue damage and chronic inflammation through cytokine-mediated and cytotoxic mechanisms (111). Furthermore, NK cell-derived IFN-γ can enhance the activation of Th1 and Th17 cells, linking the innate and adaptive immune responses in OA. Activated NK cells may also indirectly influence osteoclast differentiation by modulating the local cytokine milieu, contributing to subchondral bone remodeling (112). NK cells can interact with synovial fibroblasts, promoting the production of proinflammatory mediators such as IL-6 and TNF-α, which exacerbate joint inflammation. Persistent NK cell activation may amplify oxidative stress within the synovium, further damaging cartilage and surrounding tissues. NK cell-mediated cytotoxicity may target stressed chondrocytes, contributing to cartilage degradation and joint dysfunction. Furthermore, emerging evidence indicates that NK cells mainly amplify the IL-1β/IL-18 inflammatory axis rather than act as independent contributors to OA progression. Bridging innate and adaptive immunity, NK cells represent a potential therapeutic target for controlling inflammation and tissue damage in OA (113).

## 7 Pharmacological strategies targeting the IL-1β-IL-18 axis to preserve joint integrity

Various pharmacological strategies target the inflammatory axis (IL-1β/IL-18) to limit cartilage breakdown and maintain joint integrity in OA. The primary focus is on cytokines (IL-1β/IL-18) that are activated via caspase-1-dependent cleavage following NLRP3 inflammasome activation, thereby intensifying synovitis and degrading the extracellular matrix (ECM) (114). Currently, a range of targeted therapies targets the inflammatory axis, aiming to influence disease progression by addressing upstream regulators and pathways, as well as downstream inflammatory cascades. Key pharmacological approaches involve agents such as anakinra, canakinumab, and rilonacept that target IL-1 pathways. Anakinra is a recombinant IL-1 receptor antagonist that competitively inhibits IL-1 binding to its receptor. Canakinumab is a monoclonal antibody that neutralizes IL-1β and prevents its interaction with the IL-1 receptor. Rilonacept acts as a soluble decoy receptor in the IL-1 trap, thereby inhibiting receptor-mediated inflammatory signaling. These involve blocking the IL-1 receptor, thereby suppressing MMPs, ADAMTs, and proinflammatory cytokines. Current therapies targeting the IL-1β/IL-18 axis range from clinically evaluated cytokine inhibitors to experimental inflammasome-targeted approaches and emerging gene-based and drug-delivery technologies. The level of evidence supporting these interventions varies considerably, highlighting the need for critical evaluation of their translational potential (115–117).

IL-18 works in concert with IL-1β to amplify the inflammatory responses observed in OA by stimulating IFN-γ production, activating macrophages, and promoting chondrocyte apoptosis. IL-18 binding protein (IL-18BP) is a natural inhibitor that neutralizes IL-18 activity by suppressing the Th-1 mediated immune response and decreasing inflammation. This attenuation of inflammatory signaling contributes to reduced cartilage erosion and degeneration (83, 118).

NLRP3 is the main upstream regulator that involves IL-1β and IL-18 activation. Various key inflammasome inhibitors include MCC950 (CRID3), OLT1177 (dapanutrile), and β-hydroxybutyrate (BHB). MCC950 blocks ATP-induced NLRP3 activation, OLT1177 is an orally administered selective inhibitor that suppresses the NLRP3 inflammasome, and BHB is an endogenous metabolite that suppresses inflammasome activation (119, 120).

Caspases are crucial for cytokine maturation and inflammation signaling. VX-765 (Belnacasan) is a specific inhibitor of caspase-1, which impedes the maturation and activation of IL-1β and IL-18. This leads to decreased production of inflammatory cytokines, inhibition of chondrocyte apoptosis, and protection against cartilage degradation. This collectively contributes to the prevention of cartilage integrity and attenuation of the disease. Caspase-1 inhibition reduces the release of DAMPs, thereby modulating extracellular matrix homeostasis (121). Natural compounds involve a multitargeted therapeutic strategy that is significantly relevant to OA management. Various natural compounds may act individually or synergistically to exert anti-inflammatory effects that help manage OA. Natural compounds can scavenge free radicals generated during OA and may modulate key pathways, including NF-κB, AGE-RAGE, and NLRP3 Activation, as well as broader cytokine networks. Natural compounds, such as curcumin, inhibit the NF-κB signaling pathway and NLRP3 activation. Resveratrol is a naturally occurring phytocompound that activates SIRT1. Quercetin reduces oxidative stress and IL-1β production. Polyherbal therapies are relatively less toxic and are used to manage chronic diseases such as OA (122, 123).

Gene- and RNA-based therapies have become sophisticated methods for OA treatment. Small interfering RNAs (siRNAs) that target proinflammatory cytokines, such as IL-1β or IL-18, can reduce their expression and the activity of downstream mediators, such as MMP-13 and ADAMTS-5. MicroRNAs (miRNAs), including miR-223, play a role in inhibiting NLRP3 inflammasome activation by lowering the maturation of IL-1β and IL-18. Furthermore, miRNAs such as miR-140 and miR-146a are known to inhibit inflammatory signaling pathways and support cartilage balance. These techniques enable precise, sequence-specific targeting of key molecular pathways implicated in OA progression. These strategies underscore the potential of gene-based interventions as personalized and disease-modifying treatments for OA (124).

Advanced drug delivery systems are crucial for improving therapeutic outcomes in OA. Intra-articular administration of therapeutic drugs directly delivers them to the joints, ensuring targeted therapy. Despite promising preclinical outcomes, the clinical translation of targeted therapies targeting the IL-1β/IL-18 axis in the management of OA remains challenging. OA progression is driven not only by inflammation but also by mechanical stress, extracellular matrix degradation, aging, and metabolic dysfunction. Therefore, therapies targeting a single cytokine pathway may provide limited benefit. Future approaches may require patient stratification, combination therapies, and simultaneous targeting of multiple pathogenic mechanisms to achieve meaningful disease modification (125, 126) (**Table 3****).**

**Table 3:** Table summarizing osteoarthritis and associated immunotherapeutic options.

Therapeutic strategy	Representative agent(s)	Target/mechanism
IL-1 receptor inhibition	Anakinra	Inhibits IL-1 receptor antagonist IL-1R1
Anti-IL-1β monoclonal antibody	Canakinumab	Inactivates IL-1β
IL-1 cytokine trap	Rilonacept	Roles as soluble decoy receptor for IL-1α/IL-1β
IL-18 inhibition	IL-18 Binding Protein (IL-18BP, Tadekinig alfa)	Inhibition of IL-18 bio-activity
NLRP3 inflammasome inhibition	MCC950, OLT1177 (Dapansutrile), β-hydroxybutyrate	Prevent NLRP3 activation and inhibit caspase-1 signaling
Caspase-1 inhibiton	VX-765 (Belnacasan)	Blocks caspase-1 enzymatic activation
RNA-based therapeutics mechanism	siRNA, miR-140, miR-146a, miR-223	Supress expression of inflammatory genes and inflammasome signaling
Natural immunomodulatory compounds	Curcumin, Resveratrol, Quercetin	Inhibit NF-κB, NLRP3 and oxidative stress pathways
Nanoparticle/hydrogel mediated delivery systems	Lipid nanoparticles, polymeric nanoparticles, hydrogels	Targeted intra-articular drug delivery
Combination immunotherapy	IL-1 inhibitors coupled with antioxidant, autophagy modulators or NLRP3 inhibitors	Simultaneous targeting of multiple inflammatory pathways

## 8 Future perspectives

Significant progress in elucidating the role of the IL-1β/IL-18 axis in OA has provided a strong foundation for therapeutic development; however, several challenges persist in clinical applications. Further research should focus on the various key directions for the precision of medicinal strategies.

**Figure 5:**
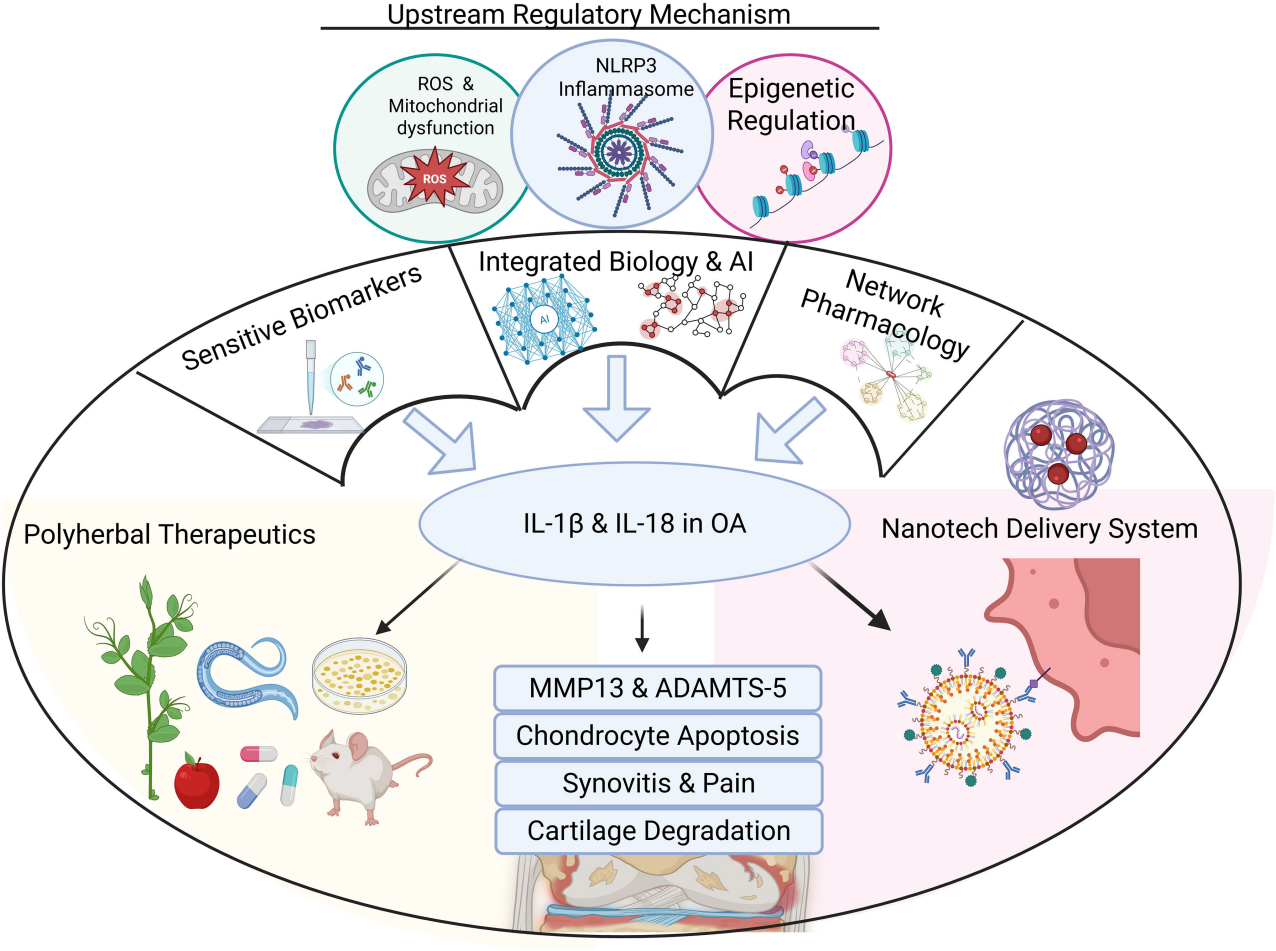
Future perspectives: IL-1β and IL-18 are key therapeutic targets for inflammation and cartilage damage in OA. Future approaches include epigenetic regulation, nanotechnology-based drug delivery systems, biomarker-guided therapy, the integration of biology and AI, and polyherbal therapeutics to slow OA progression. ROS, reactive oxygen species; AI, artificial intelligence. (Created using Bio-Render, Bio-Render license: WW29WPDN0T).

Despite substantial experimental evidence supporting the roles of IL-1β and IL-18, the clinical efficacy and therapeutic approaches of cytokine-mediated therapy in OA remain limited and underexplored. This limited finding may be attributed to the heterogeneous nature of OA, in which inflammatory mechanisms may vary across patients. Therefore, a major future challenge is to identify diverse patient groups and subgroups characterized by increased IL-1β/IL-18 activity that are most likely to benefit from targeted interventions. Furthermore, the therapeutic role of IL-18 relative to IL-1β across different stages of OA progression remains unexplored and warrants further investigation. Patient stratification based on inflammatory phenotypes and underlying molecular mechanisms may enable the development of personalized therapeutics targeting the IL-1β/IL-18 axis. This approach has the potential to improve treatment efficiency and clinical outcomes (127, 128) (**Figure 5****).**

### 8.1 Sensitive biomarkers

In early diagnostic systems, effective disease monitoring and evaluation of therapeutic responses require the development of robust and sensitive biomarkers. The upstream regulatory mechanism, particularly the modulation of inflammasomes such as NLRP3, along with various mitochondrial dysfunctions and oxidative stress, may provide broader and more effective control over cytokine activation and downstream inflammatory pathways. The clinical utility of these biomarkers remains limited due to the lack of large-scale validation and standardized assessment methodologies. Further investigations are required to validate the roles of IL-1β, IL-18, and NLRP3 inflammasome components in patient stratification, disease monitoring, and evaluation of therapeutic response (129).

### 8.2 Epigenetic regulation

Emerging evidence also points to epigenetic regulation, such as DNA methylation and histone modifications, that control IL-1β and IL-18 expression (130). OA is a multifactorial disorder; therefore, therapeutic strategies should focus on integrative, multitargeted approaches. These approaches not only focus on anti-inflammatory effects but also scavenge free radicals generated during the inflammatory process. Additionally, cartilage regeneration remains an important approach for OA management. Combination-based interventions offer a synergistic effect and improve disease-modifying ability by targeting inflammation, oxidative stress, and tissue repair, compared with single-drug approaches (14). In addition to these, a detailed understanding of the epigenetic mechanisms regulating IL-1β and IL-18 expression may reveal novel therapeutic targets, including whether epigenetic interventions can selectively suppress the pathogenic cytokine signaling pathway without disrupting normal immune mechanisms. An advanced drug delivery system may enhance the targeted delivery of therapeutic drugs in OA.

### 8.3 Nanotech delivery system

Nanotechnology-based carriers, involving lipid and polymeric nanoparticles, play an important role in targeted biological and intra-articular sustained controlled release. These systems improve drug delivery, stability, and cellular uptake, thereby minimizing adverse effects and improving the overall treatment efficacy (131). This carrier system may be particularly useful for improving intra-articular retention and, in particular, the targeted delivery of IL-1β and IL-18 inhibitors. The challenges related to long-term biocompatibility, safety, and clinical translation must be addressed to justify their application in OA treatment.

### 8.4 Integrated biology and AI

Modern therapeutic approaches rapidly integrate systems biology and artificial intelligence (AI) tools to advance OA management. The modern therapeutic application primarily integrates systems biology and artificial intelligence (AI) methods to better understand the complex regulatory networks associated with the IL-1β/IL-18 axis. These approaches mainly help in OA patient subsets with cytokine-driven disease, which predict therapeutic outcomes for further applications. Network pharmacology approaches and machine learning offer the analysis of complex cytokine interactions and their signaling networks (118, 132). This approach involves identifying potential candidate drugs that target components within the IL-1β/IL-18 signaling network. Gene-editing technologies, such as CRISPR/Cas9, also enable precise modulation of inflammasome-related genes, leading to long-term disease modification (133, 134).

### 8.5 Polyherbal therapeutics

Furthermore, polyherbal and integrative therapeutic applications, supported by scientific validation, phytocompound optimization, and mechanism-of-action studies, may provide natural, cost-effective, and safer alternatives for the long-term management of OA and its complications (134).

Various phytoconstituents have been reported to suppress NLRP3 inflammasome activation and reduce IL-1β and IL-18 production in various experimental models. While the majority of these findings remain preclinical, well-designed clinical studies are warranted to assess the efficacy, safety, and mechanisms of action in patients with OA. Concurrently, robust translation research integrating *in vitro*, *in vivo*, and clinical studies is essential, as are well-designed long-term clinical trials to assess the safety, efficacy, and durability of targeted therapies across diverse patient populations.

## 9 Limitations

OA is a mechanically driven low-grade inflammation, meaning IL-1β and IL-18 alone cannot trigger disease initiation. Various cytokines, such as TNF-α, IL-6, IL-17, and IL-36 family cytokines, act as major compensators, and blocking them can also slow OA progression. In addition, compared with IL-1β, research on IL-18 in OA has not been as extensively explored, despite growing evidence linking IL-18 to cartilage and synovial inflammation.

OA is a heterogeneous disease in which the inflammatory pathways may vary among patients and their disease stages. This heterogeneity may partly explain the variable responses observed with cytokine-targeted therapies. Another important limitation is the failure of IL-1β-targeted therapies in clinical trials for OA. Chevalier et al. reported that anakinra (IL-1 receptor antagonist) showed minimal improvement in knee OA symptoms. Another study by Ridker et al. in the Lancet showed that Canakinumab (an anti-IL-1β antibody) did not significantly modify OA progression, although it has systemic anti-inflammatory benefits. The results indicate that IL-1β functions more as a secondary amplifier cytokine rather than being the primary trigger for the onset and progression of OA (**Table 4**).

**Table 4:** Limitations of roles of IL-1β/IL-18 in OA pathogenesis.

Limitation	Explanation	References
Limited success of IL-1β clinical trials	Anakinra an IL-1 receptor antagonist showed minimal OA symptom improvement though having strong preclinical evidence	(135)
OA is not purely inflammatory driven, rather it is mechanistically driven	OA is caused by mechanical stress, ECM degradation, aging, and metabolic dysfunction before any pro-inflammatory cytokine activation	(136)
Mechanistic role of IL-18 is not as explored as IL-1β	Very few studies describe ROLE OF il-18 signalling in synovium, cartilage, and subchondral bone.	(137)
OA disease heterogeneity across patient subtypes	Metabolic related OA, age related OA, and post-traumatic OA involve different inflammatory signatures.	(138)
OA-stage specific cytokine contribution is unclear	IL-1β/IL-18 roles are different across early, intermediate, and late OA	(139)
Synovial fluid cytokine variability among patients	IL-1β expression is not consistent across OA cohorts	(140)

Furthermore, the role of IL-18 and its mechanistic and clinical studies remained unexplored, necessitating a systematic, stage-specific assessment of its role and further evaluation as a therapeutic target and biomarker for therapeutic approaches (140). Findings may suggest that IL-1β functions primarily as an amplifier of inflammatory responses rather than as a key driver of OA onset. The complexity and heterogeneity of OA underscore the need for improved patient stratification and multi-target therapeutic approaches to achieve more effective disease management (141).

## 10 Conclusion

OA is a multifactorial and heterogeneous disease that is primarily influenced by the dynamic roles of IL-1β and IL-18, both of which are members of the IL-1 family and activate signaling through a MyD88-dependent mechanism. OA is a multifaceted joint condition driven by various interactions, including those associated with aging, in which pro-inflammatory cytokines, such as IL-1β and IL-18, interact with adjacent joint cells. This text outlines the process by which IL-1β and IL-18 are activated from their inactive forms, triggering signaling pathways such as NF-κB, HIFs, Wnt, and PI3K/Akt/mTOR. These pathways subsequently enhance the production of cartilage-degrading enzymes, including Runx2, MMP13, and ADAMTS4/5. The influence of IL-1β and IL-18 extends to cells, including macrophages, synoviocytes, chondrocytes, T lymphocytes, osteoblasts, and natural killer cells, contributing to the advancement of OA.

The clinical mechanistic findings for OA remain challenging due to the biological heterogeneity of OA, which involves multiple compensatory cytokine networks, and the limited use of IL-1β-targeted therapies in clinical trials. The precise stage-specific role of IL-18 and its potential utility as a therapeutic target and biomarker require further investigation. The combined effects of IL-1β and IL-18 on joint health elucidate why targeting a single anti-inflammatory pathway is ineffective and why OA remains persistent and unresolved. Future therapeutic strategies should focus on improved patient stratification, biomarker validation, and multi-target therapeutic approaches to manage OA phenotypes.
